# Differential Effects of Monounsaturated and Polyunsaturated Fats on Satiety and Gut Hormone Responses in Healthy Subjects

**DOI:** 10.3390/foods8120634

**Published:** 2019-12-03

**Authors:** Lijuan Sun, Hui Jen Goh, Priya Govindharajulu, Melvin Khee-Shing Leow, Christiani Jeyakumar Henry

**Affiliations:** 1 Clinical Nutrition Research Centre (CNRC), Singapore Institute for Clinical Sciences (SICS), Agency for Science, Technology and Research (A*STAR), 30 Medical Drive, Singapore 117609, Singapore; lijuan_sun@sics.a-star.edu.sg (L.S.); goh_hui_jen@sics.a-star.edu.sg (H.J.G.); priya_govindharajulu@sics.a-star.edu.sg (P.G.); melvin_leow@sics.a-star.edu.sg (M.K.-S.L.); 2 Department of Endocrinology, Tan Tock Seng Hospital, Singapore 308433, Singapore; 3 Cardiovascular and Metabolic Diseases Program, Duke-NUS Medical School, Singapore 169857, Singapore; 4 Lee Kong Chian School of Medicine, Nanyang Technological University, Singapore 639798, Singapore; 5 Department of Biochemistry, Yong Loo Lin School of Medicine, National University of Singapore, Singapore 17599, Singapore

**Keywords:** high-fat meals, fat saturation, hormone response, satiety

## Abstract

The difference between fat saturation on postprandial hormone responses and acute appetite control is not well understood. The aim of this study was to compare the postprandial ghrelin, gastric inhibitory polypeptide (GIP) and glucagon-like peptide-1 (GLP1) response and subjective appetite responses after isoenergetic high-fat meals rich in either monounsaturated (MUFAs) or polyunsaturated fatty acids (PUFAs) in healthy Chinese males. A randomized, controlled, single-blinded crossover study was conducted in 13 healthy Chinese men. Two high-fat meals (64% of energy) rich in MUFAs or PUFAs were tested. Total ghrelin, GIP and active GLP1 and visual analog scale (VAS) were measured over 4 h. Ghrelin was reduced greater after MUFA compared to PUFA at the beginning of the meal (at 30 and 60 min) and was significantly negatively correlated with subjective VAS for preoccupation for both MUFA and PUFA meals. No significant difference for ghrelin 240 min incremental area under the curve (iAUCs) were found. MUFA induced higher GIP response than PUFA. GIP was associated with all the VAS measurements except preoccupation for MUFA meal. No difference was found for GLP1 between two meals, nor was GLP1 associated with VAS. In conclusion, the results demonstrate that ghrelin, GIP and VAS respond differently to MUFA and PUFA meals. Ghrelin and GIP, but not GLP1, were associated with acute appetite control, especially after MUFA meal.

## 1. Introduction

Obesity is a global epidemic and is an important risk factor for developing chronic diseases, including cardiovascular diseases (CVD), diabetes, hypertension, and dyslipidemia [1]. Body weight gain results from a chronic imbalance of energy intake and energy expenditure. A high intake of dietary fat has been implicated in the increased prevalence of obesity. This is probably caused by the increased intake of energy-dense foods and poor satiety properties of fat [2,3]. Individual fatty acids may play differential satiety roles because they vary in efficiency of absorption and rate of oxidation. 

Despite escalating number of studies comparing the effects of different composition of fatty acids on food intake or satiety, the findings were inconsistent [4,5,6,7]. Not many studies are conducted to examine the hormonal or physiological responses to dietary fatty acid composition. Satiety hormones and hunger play a vital role in appetite regulation. Ghrelin secreted by the stomach, is the only known circulating hormone that increases food intake [8]. Circulating plasma ghrelin levels prior to the ingestion of the meal are elevated to initiate feeding, followed by rapid postprandial suppression. Gastric inhibitory polypeptide (GIP) is released from duodenal endocrine K cells immediately upon the absorption of fat or glucose [9]. It promotes the efficient storage of ingested fats, and may play a pivotal role in the development of obesity due to chronic over nutrition [10]. Glucagon-like peptide (GLP1) is secreted from L cells of the distal small intestine to reduce appetite and energy intake [11,12]. Dietary manipulations that suppress ghrelin and GIP levels and increase GLP1 levels may offer pragmatic approaches for reducing energy intake. 

Many studies have been conducted to examine fatty acid based on degree of saturation and chain length diets effects on hunger and satiety signaling in the body [13,14,15,16]. Research has shown that the type of fatty acid in a meal of a diet can alter physiological and metabolic responses, although the mechanisms behind these changes are still being explored [13]. Recently, there was a paper studying the effect of MUFA (high-fat meals rich in monounsaturated), PUFA (high-fat meals rich in polyunsaturated) and SFA (high-fat meals rich in saturated) on satiety and hormone of obese women [6]. The study demonstrated that fatty acid composition differentially affected physiological markers of hunger and satiety like ghrelin and peptide YY level. However, it was unable to show changes in GLP1 level and subjective appetite ratings when alterations were made to fatty acid composition from an acute HF meal in women with obesity. In our previous study, we found that glucose and insulin responses were not affected by the degree of saturation of dietary fatty acids [17]. The aim of the present work was to compare the effect of dietary fatty acids (MUFA and PUFA) on markers of hunger and satiety (total ghrelin, GIP and GLP1), subjective feelings of satiety and the association between hormone releases and subjective satiety in Asian Chinese lean males.

## 2. Materials and Methods 

### 2.1. Subjects

Thirteen healthy Chinese men (age: 22–39 years; BMI: 18.3–28.6 kg/m^2^) participated in this study. All subjects were healthy and none had a family history of either type 2 diabetes or cardiovascular disease. None of the subjects smoked or used tobacco products, consumed special diets, or took medication known to alter metabolism. All subjects had normal fasting blood glucose concentrations and no history of food intolerance. The present study was conducted according to the guidelines laid down in the Declaration of Helsinki, and all procedures were approved by the Domain-Specific Review Board of National Healthcare Group, Singapore (2014/00719). Written informed consent was obtained from all subjects before participation. The trial was registered at clinicaltrials.gov as NCT02585427.

### 2.2. Study Design

This was a randomized, crossover, single-blinded study with two experimental trials separated by a 1-week washout period to minimize any carryover effects. On the evening before each study day (~18:30 h), subjects consumed a standardized evening meal consisting of rice, chicken, and a non-alcoholic beverage. They were then asked to refrain from consuming any foods except water until they reported to the laboratory the next morning. Vigorous physical activity was also not allowed the day before the study.

For each trial, subjects arrived at the Clinical Nutrition Research Centre (CNRC) at 08:30 h in the morning. After a 10 min rest, a 100 mm basal visual analog scale (VAS) score was determined. Thereafter, an indwelling catheter was inserted into a forearm vein by a registered nurse and a baseline blood sample was obtained (time = 0). Subsequently, subjects consumed the test meal at a comfortable pace (12 min on average). When subjects have finished consuming the test meal, they were asked to rate overall liking of the test meal. Subjective appetite ratings and venous blood samples were collected at 15, 30, 45, 60, 90, 120, 150, 180, 210, and 240 min following the start of the meal. Plasma was isolated and stored at −80 °C until further analysis. Subjects remained seated throughout the postprandial period. 

### 2.3. Satiety

The VAS tool measured subjective satiety at baseline and subsequent 10 time-specific intervals post meal. The appetite rating questionnaire included questions on hunger, desire to eat, how much food you could eat, fullness and preoccupation with thoughts of food. Scores for satiety feelings were measured with 100 mm VAS anchored at the low end with most negative or lowest intensity feelings (e.g., not at all hungry, nothing at all), and with opposing terms at the high end (e.g., extremely hungry, the most that I have ever eaten). Subjects were instructed to read each question carefully every time and indicate on a line which best reflected their feelings at that moment.

### 2.4. Test Meals

Subjects were given 2 isocaloric white rice-based meals. All meals contained 50 g of available carbohydrate cooked with 40 g of dietary fat in a rice cooker. The test meals consisted of white rice (jasmine rice) cooked with either 44 g of olive oil (monounsaturated fatty acid (MUFA)), or 40 g of grapeseed oil (polyunsaturated fatty acid (PUFA)). Fatty acid composition of two high-fat meal was shown in Table 1. The rice and dietary fat were bought from a local supermarket, and the calorie and nutrient contents of the test meals were calculated based on the weight of the components and the dietary information provided by the manufacturer. The test foods were freshly prepared in the morning on the test days. 

### 2.5. Hormone Assays

Venous blood samples were collected in BD Vacutainer^®^ containing spray-dried K2EDTA. Proteases inhibitor cocktail (cOmplete™ Mini EDTA-free, Roche, California, CA, USA) and DPP-IV inhibitor (Ile-Pro-Ile, Sigma, California, CA, USA) were added to the tubes for total ghrelin, total GIP and active GLP1 analysis. The tubes were centrifuged at 1500× *g* for 10 min at 4 °C within 15 min of sample collection. Plasma was aliquoted into eppendorf tubes and stored at −80 °C, until measurements of the hormones were performed.

Plasma total ghrelin and total GIP concentrations were measured by Elisa kits from Millipore [18]. The intra-assay CV for GIP and ghrelin were 1.8% and 3.6%. Plasma active GLP1concentrations were determined by using the human metabolic hormone MILLIPLEX MAP kit (Millipore, Cat. #HMHEMAG-34K, Missouri, MO, USA [19]. The intra-assay CV for GLP1 was 12%. The inter-assay CV for ghrelin, GIP and GLP1 were less than 10%.

### 2.6. Statistical Analysis

Statistical analysis was performed using SPSS software version 23 (IBM/SPSS Inc., Chicago, IL, USA). We estimated that a sample size of 13 subjects would allow us to detect a difference of 15% in postprandial ghrelin iAUC (our main outcome measure) between experimental meals, at α = 0.05 with a power of 80% (type II error, β = 0.2). Differences in the concentrations of ghrelin, GIP and active GLP1 were evaluated using repeated measures 2-factor ANOVA, with main effects for fat type (MUFA vs. PUFA), and time (over 240 min postprandial), as well as their interactions. The incremental areas under the curve (iAUC) were used as postprandial summary measures and were calculated by using the trapezoidal rule; data were analyzed using repeated measures 1-factor ANOVA to evaluate differences between trials at each time point. Pearson’s correlation were performed to determine associations between hormones and VAS scores. Data are presented as means ± SEM, unless otherwise stated. A *p*-value < 0.05 was considered statistically significant.

## 3. Results

All subjects completed the study and ingested the test meals without any problems; overall liking did not differ among the test meals. 

Thirteen healthy Chinese male completed the two study visits. Baseline characteristics are presented in Table 2. There were no significant changes in all the characteristics between the two visits.

### 3.1. Hormone Responses

**Figure 1:**
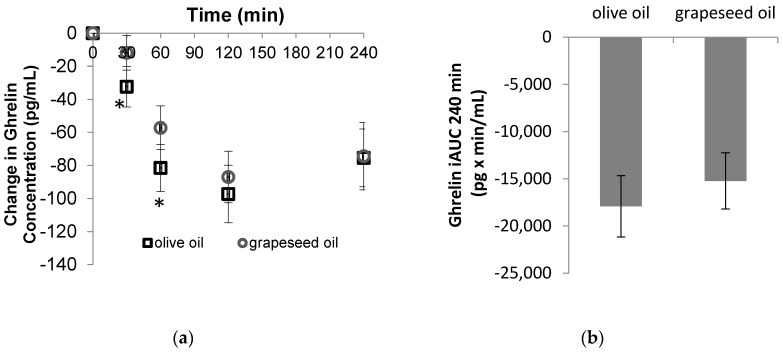

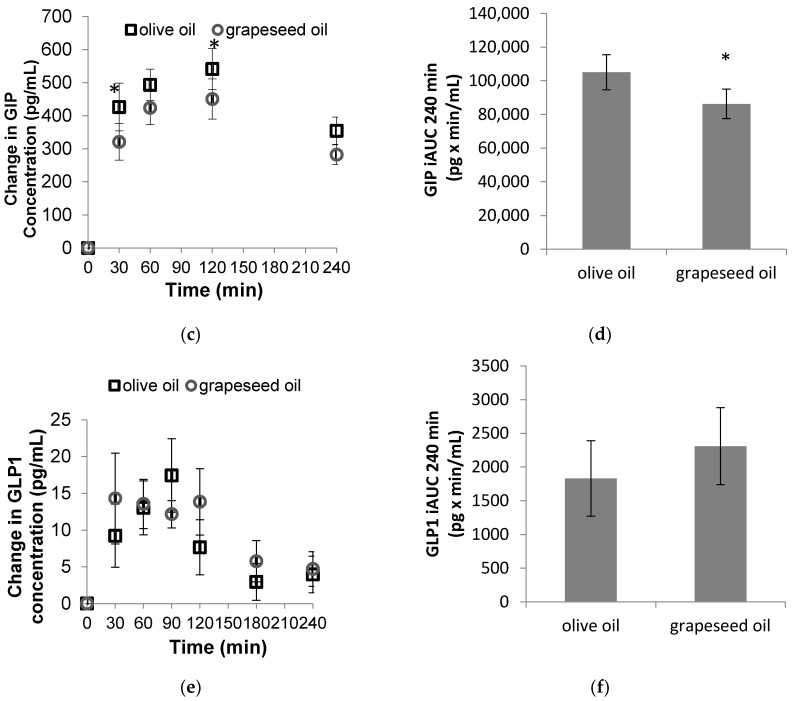
Postprandial plasma total ghrelin (**a**), gastric inhibitory polypeptide (GIP) (**c**) and active glucagon-like peptide-1 (GLP1) (**e**) concentrations (change from fasting values) and ghrelin (**b**), GIP (**d**) and active GLP1 (**f**) incremental areas under the curve (iAUC) for 240 min between olive oil and grapeseed oil ingestion with white rice. * Indicates significance at *p* < 0.05 between two treatments. All values are mean ± SEM, total *n* = 13.

The postprandial time course of meal responses for change from baseline in plasma ghrelin, GIP, active GLP1 for two treatment meals are shown in Figure 1. For change in ghrelin, there was a significant of time (*p* < 0.001) effect but not fat type (*p* = 0.094) and fat type by time interaction effect (*p* = 0.139). However, MUFA had a greater reduction in ghrelin compared to PUFA at 30 min and 60 min (Figure 1a). No significant difference was found for 240 min iAUC (Figure 1a). However, the 120 min iAUC for the ghrelin response was significantly higher after PUFA meal than after MUFA meal. For change in GIP, there was a significant main effect for fat type (*p* = 0.031) and time (*p* < 0.001) but no significant fat type by time interaction (*p* = 0.185). GIP concentrations at 30 min and 120 min were significant higher after the MUFA rich meal compared to the PUFA rich meal (Figure 1b). The 240 min iAUC for the GIP response after MUFA meal was significantly higher than after of PUFA meal (Figure 1b). No significant difference in the postprandial active GLP1 was found between the two fat rich meals (Figure 1c).

### 3.2. Subjective Satiety Responses

**Figure 2:**
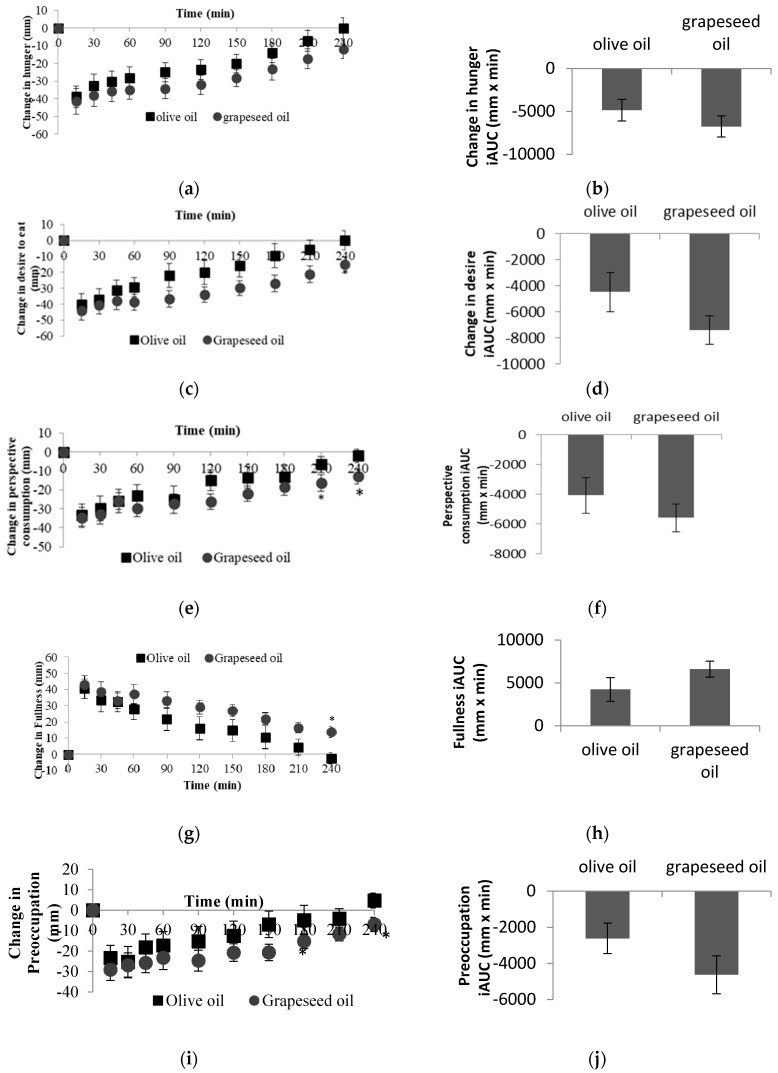
Postprandial satiety VAS markers hunger (**a**), desire to eat (**c**), perspective consumption (**e**), fullness (**g**) and preoccupation with thoughts of food (**i**) (change from fasting values) and hunger (**b**), desire to eat (**d**), perspective consumption (**f**), fullness (**h**) and preoccupation with thoughts of food (**j**) incremental areas under the curve (iAUC) for 240 min between olive oil and grapeseed oil ingestion with white rice. * Indicates significance at *p* < 0.05 between two treatments. All values are mean ± SEM, total *n* = 13.

4 h postprandial subjective VAS measures for hunger, desire to eat, fullness and preoccupation with thoughts of food ratings were shown in Figure 2. Both of the meals significantly suppressed hunger, desire to eat and preoccupation with thoughts of food and increased fullness (time effect *p* < 0.001 for all VAS markers). In general, changes in hunger was not different between two meals (Figure 2a). Grapeseed oil induced a greater suppression in change in desire to eat at 240 min post meal compared with olive oil (Figure 2c). Two high-fat meals related changes in how much subjects wanted to eat (prospective consumption) and preoccupation with thoughts of food over 4 h suggested greater suppression with grapeseed oil at 210 min and 240 min post meal (Figure 2e,i). Change in rating of fullness was higher after consuming high-fat meal containing grapeseed oil than olive oil at 240 min post meal (Figure 2g). In summary, PUFA-rich meal elicited greater fullness and a lower “desire to eat”, “perspective consumption” and “preoccupation” ratings compared with MUFA-rich white rice meal at 240 min.

### 3.3. Correlations

Correlations for 240 min iAUCs for satiety versus in hormones were shown in Table 3. For ghrelin, there was a significant negative correlation with preoccupation with thoughts of foods iAUC for both MUFA and PUFA meals. Significant positive correlations were observed between GIP and “hunger”, “desire to eat” after MUFA meal. There was a significant negative correlation between GIP and VAS fullness iAUC for MUFA meal. No correlations for GLP1 versus any satiety scores for both of the fats were found.

## 4. Discussion

The present study aimed to evaluate the effects of degree of unsaturation of fats in a meal on postprandial satiety markers in healthy Chinese male subjects. Two meals were isocaloric and had the same macronutrient composition, and differed only in the degree of unsaturation of the fat. Our results showed that postprandial ghrelin and GIP responses are affected by the degree of saturation of dietary fat, with olive oil (MUFA) resulting in greater postprandial GIP concentration and lower ghrelin concentration as compared to grapeseed oil (PUFA). However, MUFA- and PUFA-rich white rice based meal had effects on subjective measures only at the end of the study. These findings indicate that 40 g of oil in the meal may change the physiological satiety markers earlier than the change of the subjective satiety levels. 

It has been reported that energy from fat has a weaker effect on satiety than isoenergetic amount of carbohydrate [20] and protein [21]. Thus, high-fat foods tend to promote overconsumption and lead to weight gain and obesity [22]. Besides the palatability and high energy density of dietary lipids, the quality and type of fat may be another important factor causing weight gain. Consumption of different types of fat has been associated with different rates of weight gain [23]. Chain length and degree of saturation of fatty acids are the two prominent features which may affect fat oxidation and possibly energy expenditure [24]. To better understand the relationship between dietary fatty acid composition and satiety in humans, three different measurement approaches have been used in previous studies. Some researchers focused on the physiological response by measuring hormones changes known to affect hunger or satiety [6,25]. Some researchers conducted questionnaires such as VAS to examine subjective feelings of hunger and fullness [26,27]. Others may examine satiety by measuring energy or food intake at subsequent meals [5,28]. In our current study, we measured several gut hormones and VAS alteration to compare the two types of fat (MUFA vs. PUFA) effects on hunger or satiety.

While chain length of dietary fatty acids has been reported to have different effects on satiety hormones [29,30], less is known about the hormonal responses based on the degree of saturation in fatty acids. Different gastrointestinal system hormones have been found to play different roles in the regulation of satiety after consumption of different fatty acid-rich meals. In our study, we selectively measured postprandial ghrelin, GIP and GLP1 response after MUFA and PUFA rich meals. 

Ghrelin, known as the hunger hormone, is released primarily from the fundus of the stomach and binds to its receptors to stimulate food intake [31]. Dietary ghrelin is a physiological mediator of feeding [32]. In fasting state, the ghrelin reaches higher levels and then decreases immediately after food consumption. Ghrelin level was reduced significantly more after MUFA-rich meal compared with PUFA rich-meal soon after meal consumption. This suggests stronger satiety was induced after MUFA in the early stage. However, the ghrelin iAUC was not different between MUFA and PUFA rich meal throughout the whole study period. The suppressive effect of MUFA rich meal seems to diminish over time. Therefore, it remains unknown whether this acute difference of ghrelin change in the short period we observed could affect long term satiety. 

There is convincing evidence that GLP1 is one of the meal-induced satiety response mediators. GLP1 inhibits gastric emptying and the activity of the central nervous system involved in the regulation of satiety [33,34]. Active GLP1 level increased after both MUFA and PUFA meals. We did not find any difference in GLP1 level between treatments which was consistent with the previous study [6]. However, it contradicts the results of previous studies that MUFA meal induced higher GLP1 [25,35]. The discrepant results between different studies may be due to the fat amount, subjects’ body composition and ethnicity. 

GIP is the second key incretin hormone which is involved in the regulation of satiety [36]. In contrast to GLP1, the postprandial GIP level is inversely related to the subsequent feeling of satiety [37,38]. In our study, a higher postprandial GIP response after ingestion of a high-fat test meal was found and this is consistent with results from a previous study [39]. In addition, MUFA induced a significantly higher postprandial GIP response compared to PUFA which suggested that PUFA may exert a relatively higher satiety control. 

The effect of degree of unsaturation of fatty acids on subjective feelings of hunger and fullness has been studied [4,40]. Most of the studies did not find any significant effects of dietary fatty acids on subjective feelings of hunger and fullness [4,5,28]. Similarly, we did not find any significant treatment effect for the VAS questions. However, we found that PUFA-rich meal elicited greater fullness and a lower “desire to eat”, “perspective consumption” and “preoccupation” ratings while MUFA-rich white rice meal showed weaker effects on those subjective feelings questions at the end of the study (at 240 min). This is in line with previous report [27] which showed PUFA elicited a relatively stronger control over appetite than MUFA. The ratings of hunger was not significantly different at 240 min between the two high-fat meals which is consistent with the previous study [4]. This is probably due to a slightly bigger variation between subjects for their response to hunger and also a smaller sample size compared with the other study [27]. 

GIP response was found significantly correlated with VAS scores only after MUFA-rich meal which suggested GIP could translate to differences in the feelings of hunger, fullness, or desire to eat. Interestingly, ghrelin iAUC at 240 min of the study was negatively correlated with only preoccupation with thoughts of food VAS score. VAS is a psychometric response scale in general, and is used to measure hunger and satiety subjectively which suggested to be consistent within human subjects [13]. However, based on our results, although the five VAS scores changed in consistent manner, it seems preoccupation with thoughts of food related with postprandial ghrelin strongly after both MUFA and PUFA rich meals. The discrepancies between VAS data and hormone results measured from different studies raise an important question as to whether VAS lacks a precise and consist match between physiologic and psychologic appetite data. 

## 5. Conclusions

In summary, after food consumption, the ghrelin level expectedly decreased with decreased hunger, while GLP1 and GIP levels increased with increased satiety. The exploration of more molecular hormone markers such as leptin, cholecystokinin (CCK), peptide YY (PYY), etc. may help to better correlate with subjective data. Taken together, the results of the study suggested that MUFA may exert a relatively weaker effects on appetite compared with PUFA. The type of fatty acid in a diet can alter physiological and metabolic responses, the mechanisms behind these changes are still needed to further explore.

## Figures and Tables

**Table 1:** Fatty acid composition of test meals with olive oil and grapeseed oil.

	Olive Oil	Grapeseed Oil
Amounts per Serving (g)	44.4	40.0
Calories (kcal)	364.0	360.0
Total fat (g)	40.4	40.0
Saturated Fat (g)	6.1	4.0
Monounsaturated fat (g)	31.3	7.6
Polyunsaturated fat (g)	3.0	28.4

**Table 2:** Baseline participant characteristics.

Measurements	Average	SD
Age (year)	27.1	6.4
Weight (kg)	70.8	10.3
Height (cm)	174.4	4.5
Body mass index (kg/m^2^)	23.3	3.2
Body fat percent (%)	19.1	5.1
Waist circumference (cm)	79.7	9.3
Hip circumference (cm)	96	7.2
Waist-to-hip ratio	0.89	0.05

**Table 3:** Correlations between iAUC 240 min of hormones and iAUC 240 min in visual analog scale measurements.

	**iAUC240**	**Hunger**		**Desire**		**Fullness**		**Quantity**		**Preoccupation**	
		r	*p*	r	*p*	r	*p*	r	*p*	r	*p*
MUFA	GIP	0.57 *	0.04	0.58 *	0.04	−0.57 *	0.04	0.59 *	0.03	0.22	0.47
	Ghrelin	−0.13	0.66	−0.18	0.57	−0.07	0.82	0.03	0.92	−0.58 *	0.04
PUFA	GIP	0.01	0.98	0.17	0.59	−0.01	0.99	−0.07	0.83	−0.03	0.92
	Ghrelin	−0.33	0.27	−0.30	0.31	0.19	0.53	−0.18	0.57	−0.57 *	0.04

* Indicates significance at *p* < 0.05. MUFA = monounsaturated fatty acid; PUFA = polyunsaturated fatty acid; GIP = Gastric inhibitory polypeptide; iAUC = incremental area under the curve.
